# FALL RISK AND FEAR OF FALLING IN LOW-INCOME COMMUNITY-DWELLING OLDER ADULTS USING A SIMPLE CHECKLIST

**DOI:** 10.1093/geroni/igad104.3347

**Published:** 2023-12-21

**Authors:** Aleeyah Uddeen, Renata Komalasari, Ladda Thiamwong

**Affiliations:** University of Central Florida, Orlando, Florida, United States; Penn State Nursing, State College, Pennsylvania, United States; University of Central Florida, Orlando, Florida, United States

## Abstract

Fall risk is common among older adults and can seriously threaten their well-being and increase fear of falling (FOF). FOF can affect the ability of older adults to maintain daily activities and may cause depression. Using a simple checklist to assess the levels of fall risk and FOF can help tailor interventions to prevent falls and promote physical activity. The purpose of the study was to assess fall risk and FOF using simple checklists and examine the association between fall risk and FOF in community-dwelling older adults in low-income settings. A descriptive cross-sectional design was used to assess 103 older adults (men=16, women=87, mean age=75.70, SD±7.19). We used the CDC’s Stay Independent checklist with twelve items to measure fall risk and the Short Fall Efficacy Scale International (Short FES-I) with seven items to assess FOF. Scores equal to or greater than four on the Stay Independent and greater than ten on the Short FES-I indicate high fall risk and high FOF, respectively. The data was analyzed using SPSS version 28. Spearman correlation tests showed that fall risk was positively correlated with FOF (r=.606, p<.001). Our finding suggests that using simple checklists to assess both fall risk and FOF may be helpful for older adults in understanding their risk of falls, thus attenuating their FOF. It may be useful to increase accessibility to these two checklists in efforts to prevent falls and FOF among community-dwelling older adults in low-income settings.

